# Towards Robust Thermal MEMS: Demonstration of a Novel Approach for Solid Thermal Isolation by Substrate-Level Integrated Porous Microstructures

**DOI:** 10.3390/mi13081178

**Published:** 2022-07-26

**Authors:** Ole Behrmann, Thomas Lisec, Björn Gojdka

**Affiliations:** Fraunhofer-Institute for Silicon Technology ISIT, 25524 Itzehoe, Germany; thomas.lisec@isit.fraunhofer.de (T.L.); bjoern.gojdka@isit.fraunhofer.de (B.G.)

**Keywords:** porous, thermal, isolation, insulation, MEMS, sensor, infrared, flow, heat transfer

## Abstract

Most current thermal MEMS use fragile structures such as thin-film membranes or microcantilevers for thermal isolation. To increase the robustness of these devices, solid thermal insulators that are compatible with MEMS cleanroom processing are needed. This work introduces a novel approach for microscale thermal isolation using porous microstructures created with the recently developed PowderMEMS wafer-level process. MEMS devices consisting of heaters on a thin-film membrane were modified with porous microstructures made from three different materials. A thermal model for the estimation of the resulting thermal conductivity was developed, and measurements for porous structures in ambient air and under vacuum were performed. The PowderMEMS process was successfully used to create microscale thermal insulators in silicon cavities at the wafer level. Measurements indicate thermal conductivities of close to 0.1 W/mK in ambient air and close to 0.04 W/mK for porous structures under vacuum for the best-performing material. The obtained thermal conductivities are lower than those reported for both glass and porous silicon, making PowderMEMS a very interesting alternative for solid microscale thermal isolation.

## 1. Introduction

Thermal MEMS rely on the generation and/or absorption of heat at the microscale. Following simple resistive heaters, the three main classes of thermal MEMS are sensors, actuators, and energy harvesters. In the field of sensors, applications span from the detection of infrared radiation (IR) to calorimetric flow sensors, thermal accelerometers, and a variety of different gas-sensing principles [1,2,3,4,5,6]. Thermal actuators rely on the controlled deformation of a MEMS structure upon heating and cooling of defined areas. The main applications are as micromechanical switches and as tilt-actuators for micromirrors [7,8]. Thermal MEMS energy harvesters exploit the thermoelectric or pyroelectric effect to generate electrical energy from temperature gradients to power low-energy devices such as wearable sensors or implants [9].

Most thermal MEMS require the confinement of heat generation or absorption in a well-defined area to function. This necessitates the use of thermal isolation strategies that are, however, not trivial to implement due to the high thermal conductivity of monocrystalline silicon. The most common approach is to remove as much silicon as possible around the thermally active microstructure, which is mostly a metal thin-film. This can be achieved by creating thin-film membranes, suspension by microcantilevers, or the complete removal of any substrate leaving only the free-standing metallization [10]. All these approaches have the drawback that they create fragile structures with MEMS processes that are often difficult to control, as film stresses need to be carefully adjusted to prevent buckling or outright failure of the structures. Furthermore, suspended microstructures and thin membranes are sensitive to damage by vibration or pressure shocks, limiting their use in rough environments.

The previously mentioned challenges have led MEMS designers to consider the use of thermally isolating bulk materials. The two main materials are glass and porous silicon. Both materials offer thermal conductivities that are more than two orders of magnitude lower than that of monocrystalline silicon [11,12,13]. Although suitable for some applications, these values are still much larger than the thermal conductivities that can be achieved with structures such as thin-film membranes or cantilevers [14,15].

Recently, PowderMEMS, a novel back-end-of-line (BEOL) compatible process for the creation of porous microstructures at the wafer level, has been described [16]. In brief, the process begins with the introduction of a dry loose powder into microcavities formed by, e.g., deep reactive ion etching (DRIE) [17]. Atomic layer deposition (ALD) is then used to agglomerate the loose powder in situ. Finally, the wafers are cleaned of any unwanted powder residues and are ready for further processing under standard MEMS cleanroom conditions. In previous work, the use of PowderMEMS structures for energy harvesting and zero-powder wakeup [18,19], permanent micromagnets and magnetic position detection [20,21], and the creation of liquid-cooled microscale inductor cores [22] has been presented.

In this work, a novel approach for the thermal isolation of MEMS components based on PowderMEMS microstructures is presented. The process was used to create porous structures at the wafer level inside microcavities beneath thin-film membranes with embedded heaters. A thermal model was developed to estimate the resulting thermal conductivities of the structures. Since the thermal conductivity of porous structures is strongly reduced once the mean free path length of the gas inside the structure approaches the pore size (Knudsen effect) [23], measurements were performed both in ambient air and under vacuum.

## 2. Materials and Methods

### 2.1. Sensor Layout

**Figure 1:**
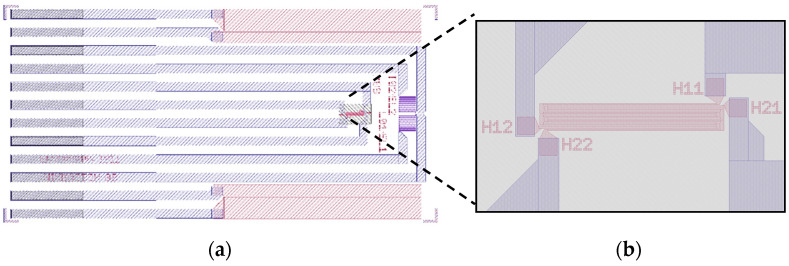
(**a**) Overview of the layout of the sensor. The outer dimensions of the die are 12 mm × 6 mm. (**b**) Detailed view of the heater structure (red) and the membrane area (grey, 890 µm × 550 µm). Only this structure is used in this work.

The devices used in this work were originally designed as multipurpose sensors to measure flow, temperature, and conductivity in drinking water [24]. Figure 1a gives a general overview of the sensor design, including all active electrodes and the connecting traces. In this work, only the structure originally designed to work as a calorimetric flow sensor was used. The structure consists of two intertwined thin-film molybdenum (Mo) heaters with electrical connections facilitated by an AlCu_0.5_ thin film. Depending on the substrate used for processing, these heaters are located either on a thin-film membrane (blue area in Figure 1b) or directly on the substrate.

### 2.2. Sensor Fabrication

**Figure 2:**
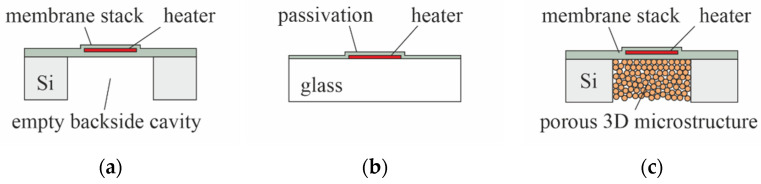
Test structures as fabricated on silicon or glass wafers: (**a**) heater embedded within a free-standing membrane; (**b**) heater with passivation on glass; (**c**) heater embedded within a membrane on top of a porous 3D microstructure for thermal isolation.

All sensors use the same frontside layout (Figure 1) and are fabricated on 725 µm thick, 8-inch silicon, or Borofloat 33 glass wafers (Figure 2). The frontside thin-film stack comprised of passivation layers, a heater, and metal traces with bond pads (not shown in Figure 2), is always manufactured using the same mask set and the same materials. In the case of silicon-based sensors, an additional mask is used to define the regions for backside etching.

**Figure 3:**
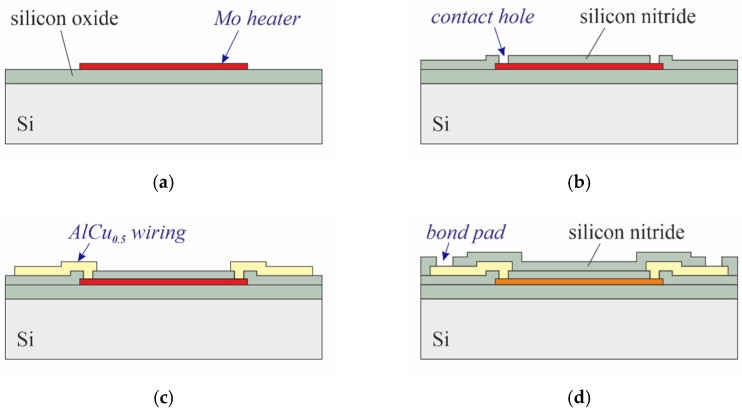

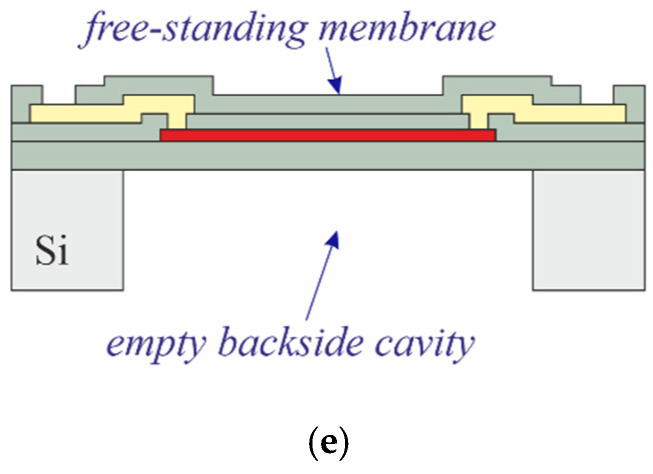
Fabrication of the sensor on silicon.

On silicon substrates, the fabrication of the devices starts with the deposition of 5 µm silicon oxide by plasma-enhanced chemical vapor deposition (PECVD), acting as electrical and thermal isolation to the substrate. Then, 150 nm molybdenum (Mo), the material for the heaters, is sputtered. After the 1st lithography, the Mo is patterned by reactive ion etching (RIE) followed by resist removal and surface cleaning (Figure 3a). A quantity of 1 µm of PECVD silicon nitride is then deposited to seal the Mo pattern. After the 2nd lithography, contact holes to the Mo layer are created by RIE (Figure 3b). Next, sputtering of 1 µm AlCu_0.5_ is followed by the 3rd lithography and the patterning of the metal by RIE (Figure 3c). Another 0.5 µm of PECVD silicon nitride is then deposited as protection for the AlCu_0.5_ traces. After the 4th lithography, the bond pads are exposed by RIE. The total thickness of the PECVD-Si_3_N_4_ passivation above the Mo heaters is 1.5 µm. The cross-section in Figure 3d depicts the device after completion of all frontside processes. For backside etching, the substrate is turned upside down. After the 5th lithography (backside), the silicon substrate beneath the membrane area is completely removed by deep reactive ion etching (DRIE). Figure 3e shows a corresponding cross-section after resist stripping in O_2_ plasma.

**Figure 4:**
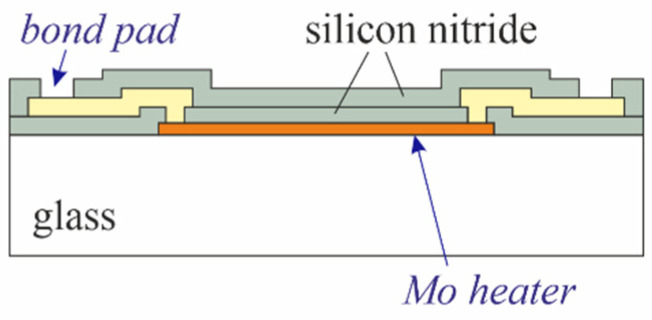
Cross-section through a finished sensor on glass.

Sensors on glass (Figure 2b) are processed in the same way as illustrated in Figure 3, but omitting both the first 5 µm PECVD silicon oxide layer and the backside processing. Figure 4 presents a schematic cross-section through the finished device.

### 2.3. PowderMEMS Modification

The three powders used in this work were “Aeroperl (AP) 300/30” a specially granulated form of pyrogenic silicon dioxide (Evonik, Essen, Germany) with $D_{50}=33\;µm$, silicon nitride (Sigma Aldrich, Schnelldorf, Germany) with $D_{50}<10\;µm$, and glassy carbon (Sigma Aldrich, Germany) with $D_{50}=2-12\;µm$.

**Figure 5:**
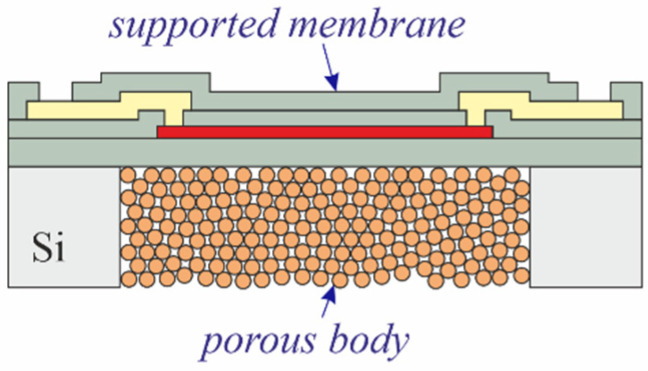
Cross-sections through finished sensor with porous PowderMEMS isolation according to Figure 2c.

To obtain test structures in accordance with Figure 2c, the frontside of the substrate is coated with photoresist and laminated with UV tape to protect it during subsequent processing. Next, the wafers are transferred from the cleanroom into the dedicated PowderMEMS lab. In this lab, loose, dry powder is filled into the backside cavities and then agglomerated into solid 3D-microstructures by atomic layer deposition (ALD) of 75 nm Al_2_O_3_ at 75 °C [16]. Figure 5 shows a cross-section through a sensor after PowderMEMS processing (Figure 2c), UV tape detachment, and photoresist stripping in O_2_ plasma.

### 2.4. Measurement Setup

**Figure 6:**
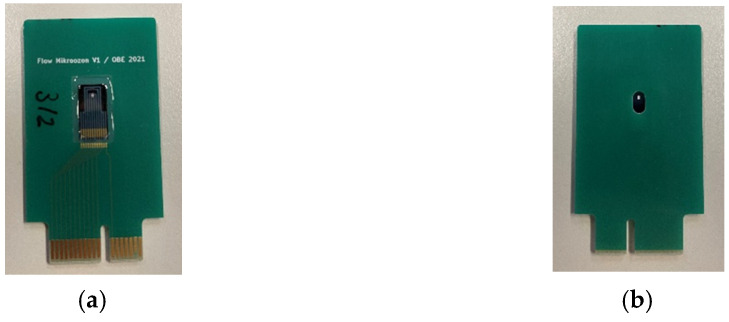
Sensor mounted on custom PCB: (**a**) top-view showing the sensor and card-edge connector and (**b**) bottom-view showing the hole below the PowderMEMS-modified backside cavity.

After dicing of the finished wafers, individual chips are mounted onto custom printed circuit boards (PCBs) (Figure 6a). The PCBs are designed with a hole located beneath the backside cavity of the chip (Figure 6b). Electrical connections from the chip to the PCB are made by ultrasonic aluminum wire bonding. Finally, a sealing compound is dispensed around the outer perimeter of the chip to ensure a vacuum-tight seal.

**Figure 7:**
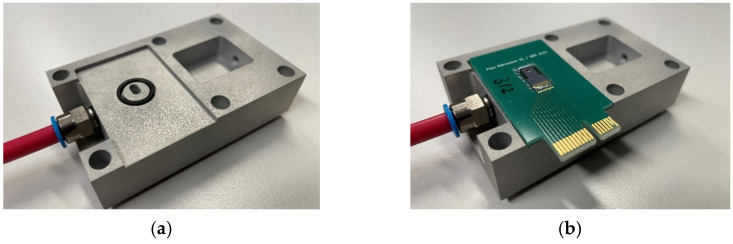
Custom vacuum chuck without (**a**) and with (**b**) mounted PCB. By connecting the pink tubing to a turbopump, a vacuum can be created inside the porous PowderMEMS structure located beneath the thin-film membrane.

Figure 7 shows a custom chuck that enables the creation of a vacuum inside the porous PowderMEMS structure. The vacuum port (Figure 7a) lines up with the hole in the PCB (Figure 7b). The port is then connected to a turbopump via plastic tubing.

Electrical measurements are performed by connecting one terminal of the thin-film heater to a controllable voltage source (LabJack U6, LabJack, Lakewood, CO, USA). The second terminal is connected to ground via a series resistor. By measuring the voltage drop across the known series resistor, the heating current is obtained. Using the calculated current and by measuring the voltage drop across the thermistor, the resistance of the thermistor is then derived using Ohm’s law.

### 2.5. Measurement Principle and Simulation Model

**Figure 8:**
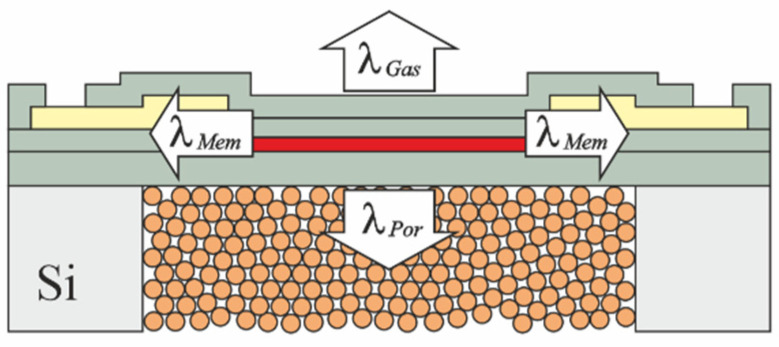
Simple 2D thermal model of the PowderMEMS-modified sensor.

Figure 8 shows a simple 2D thermal model of a heater on a thin-film membrane on top of a porous structure. If the heater is powered with a constant power $P_{0}$, it will be at overtemperature T above the ambient temperature. T is dependent on the thermal conductivities of the gas above the membrane ($\lambda_{Gas}$), the porous structure below the membrane ($\lambda_{Por}$), and the membrane itself ($\lambda_{Mem}$).

The power $P_{0}$ that is needed to maintain a constant T can thus be written as the sum

(1) $$P_{0}=G_{Gas}\lambda_{Gas}T+G_{Mem}\lambda_{Mem}T+G_{Por}\lambda_{Por}T$$

with factors G that are dependent on the individual geometry of the MEMS device [25]. By rearranging Equation (1), an expression for T in terms of the heating power and the thermal conductivities can be found as

(2) $$T=\frac{P_{0}}{G_{Gas}\lambda_{Gas}+G_{Mem}\lambda_{Mem}+G_{Por}\lambda_{Por}}$$

The thermal resistance $R_{Th}$ of the device, which is defined by the change in T with respect to $P_{0}$, can then be found by taking the partial derivative of Equation (2) with respect to $P_{0}$, yielding

(3) $$R_{Th}=\frac{\partial }{\partial P_{0}}\left(T\right)=\frac{1}{G_{Gas}\lambda_{Gas}+G_{Mem}\lambda_{Mem}+G_{Por}\lambda_{Por}}$$

Since both products $G_{Gas}\lambda_{Gas}$ and $G_{Mem}\lambda_{Mem}$ remain constant, they can be combined into a single constant K, and Equation (3) reduces to

(4) $$R_{Th}\left(\lambda_{Por}\right)=\frac{1}{K+G_{Por}\lambda_{Por}}$$

By solving Equation (4) for $\lambda_{Por}$, the final model that allows for the determination of the thermal conductivity of the porous microstructure from the measured thermal resistance $R_{Th}$ is found to be

(5) $$\lambda_{Por}\left(R_{Th}\right)=\frac{1-R_{Th}K}{R_{Th}G_{Por}}$$

**Figure 9:**
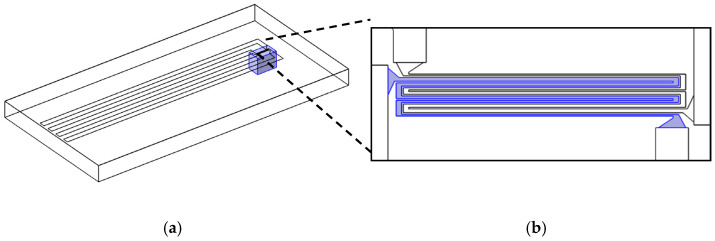
(**a**) Simulation model for the sensor structure on top of a thin-film membrane on a silicon substrate with a backside cavity. The thermal conductivity of the volume inside the cavity (blue) is $\lambda_{Por}$. (**b**) Detailed view of the heater structures. The active heater is highlighted in blue.

As the geometry factor $G_{Por}$ and the combined constant K cannot be found analytically for a complex 3D geometry such as the one used in this work, they are determined by the finite element method. For this, models representing the sensor on both a thin-film membrane within a silicon frame and directly on a glass substrate were developed in COMSOL Multiphysics (see Figure 9). All lateral dimensions correspond to the actual devices (see Figure 1). The vertical stack is simplified to aid meshing. To simulate devices manufactured on glass wafers, the silicon substrate and backside cavity are replaced by a glass domain.

In the case of the silicon model, the vertical stack consists of 725 µm monocrystalline silicon ($\lambda_{Si}=130\;W/\mathrm{mK}$) followed by 5 µm silicon oxide ($\lambda_{SiO_{2}}=1.4\;W/\mathrm{mK}$). On top of this layer, all metals are structured in a single layer of zero thickness. For simplicity, all passivation layers are combined into a single 1.5 µm silicon nitride layer ($\lambda_{Si_{3}N_{4}}=3\;W/\mathrm{mK}$) [26,27] on top of the metal layer. The thermal conductivity of the volume of the cavity under the membrane can be varied according to the value of $\lambda_{Por}$. For the glass model, the silicon oxide membrane layer is omitted, and the metal layer sits directly on top of the 725 µm thick substrate ($\lambda_{Bf33}=1.1\;W/\mathrm{mK}$) [28]. The passivation layer is the same as that of the silicon model. Boundary conditions are chosen corresponding to the experimental conditions. For the top surface, a COMSOL-provided model for conductive/convective heat flux from a horizontal plate into ambient air (296.15 K) is used. All other outer boundaries are set to be at a constant ambient temperature of 296.15 K. The heat source is realized as a boundary heat source corresponding to the geometry of one of the Mo heaters (Figure 9b). The input parameters of the model are thus the heating power $P_{0}$, which is dissipated from one of the Mo heater boundaries, and, in the case of the silicon sensor, the thermal conductivity $\lambda_{Por}$ of the porous domain inside the backside cavity. The output parameter of the model is the temperature T of the heater, which is measured as the average temperature of the heated Mo heater boundary.

## 3. Results

### 3.1. Measurement of the TCR

For the determination of the temperature coefficient of resistance (TCR) of the Mo heater structures, wafer-level measurements were carried out. The wafers were placed on a heated chuck and the electrical resistance of the heaters was recorded during a temperature sweep from 30 to 90 °C in steps of 20 °C. The average TCR of the Mo thin film in this temperature range was found to be $TCR_{Mo}=2.38\cdot 10^{-3}\;K^{-1}$, which is lower than that of the bulk material [29].

### 3.2. PowderMEMS Microsctructures

**Figure 10:**
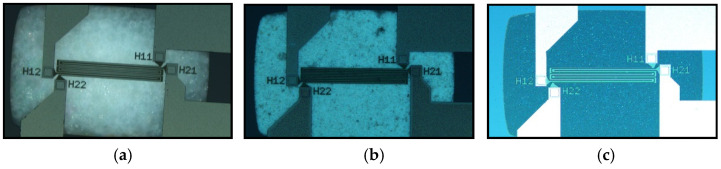
Topside micrographs of the PowderMEMS-modified devices. The solid porous microstructures are visible through the optically transparent membrane stack. (**a**) AP 300/30. (**b**) Silicon nitride. (**c**) Glassy carbon.

Figure 10 shows micrographs of the sensors with PowderMEMS microstructures inside the backside cavity. The structures are readily visible through the optically transparent membrane stack. The optically invisible ALD layer envelops and connects each particle to its neighbors, forming a solid porous structure. The ALD layer also connects the particles to the inside walls of the backside cavity, and to the underside of the membrane, leading to mechanical stabilization of the membrane.

### 3.3. Thermal Conductivity of the PowderMEMS Microstructure

As described previously, the basic strategy to obtain the thermal conductivity $\lambda_{Por}$ of the porous 3D microstructures is to measure the thermal resistance $R_{Th}$ of the sensor by applying a voltage sweep to one of the Mo heaters and then using the model presented in Equation (5) to calculate $\lambda_{Por}$. The first step is to determine the model constants K and $G_{Por}$ by 3D FEM simulation of the sensor geometry.

#### 3.3.1. Quality of FEM Simulation and Fitting of Thermal Model

To evaluate the quality of the FEM model, measurements of $R_{Th}$ were taken of unmodified (i.e., empty backside cavity) membrane sensors and sensors processed on a glass substrate in ambient air because, for these cases, the thermal conductivities of the media beneath the sensors are known. The measured values were compared with values obtained by FEM simulation. The results are presented in Table 1 and show that the simulated values closely match the measurements.

**Figure 11:**
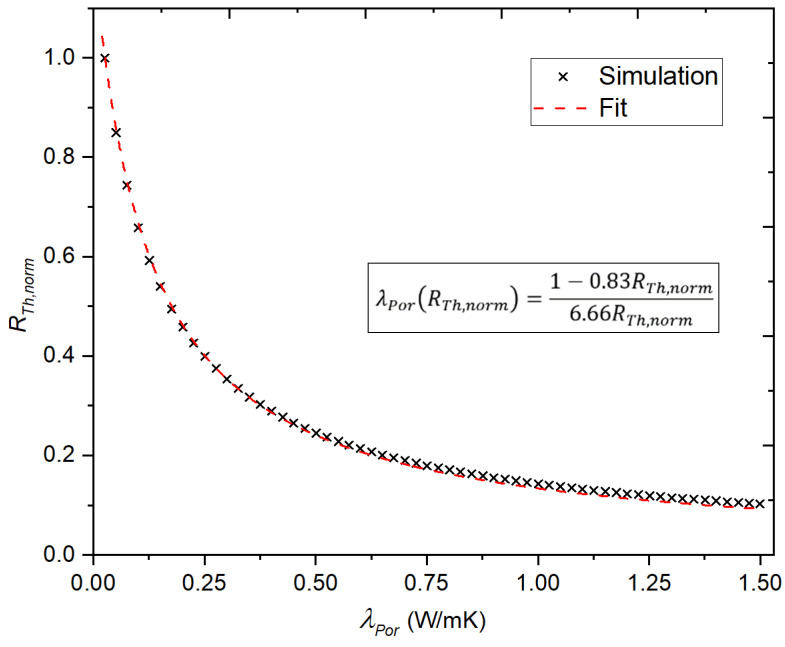
Fit of the thermal model (Equation (5)) to the normalized results of a sweep of $\lambda_{Por}$ in the interval [0.026,1.5]. The fitted parameters were found to be K=0.83 m and $G_{Por}=6.66\;m$ at $R^{2}=0.99$.

To obtain values for the model constants K and $G_{Por}$ by FEM simulation, a sweep of $\lambda_{Por}$ was carried out and the resulting values for $R_{Th}$ were calculated and normalized with respect to the value obtained for ambient air. The thermal model presented in Equation (5) was then fitted to the resulting data points, yielding values for K=0.83 m and $G_{Por}=6.66\;m$. Both the simulated data points and the resulting fitted curve are presented in Figure 11.

Using the obtained values, the thermal model presented in Equation (5) can then be written as

(6) $$\lambda_{Por}\left(R_{Th,norm}\right)=\frac{1-0.83R_{Th,norm}}{6.66R_{Th,norm}}$$

#### 3.3.2. Estimation of Thermal Conductivity

**Figure 12:**
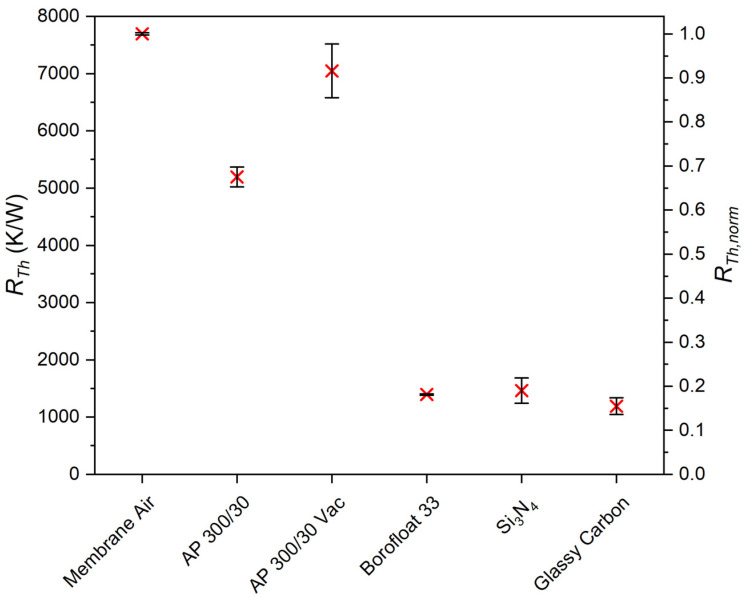
Left axis: Measurements of the thermal resistance $R_{Th}$ of the PowderMEMS-modified devices in comparison to a thin-film membrane surrounded by air and devices directly processed on Borofloat 33 glass. Right axis: Measurements normalized with respect to the measurement obtained for the thin-film membrane in air (“Membrane Air”).

The thermal resistance $R_{Th}$ was then measured for sensors modified with porous PowderMEMS microstructures. Reference measurements were taken of sensors without PowderMEMS modification and of sensors manufactured on Borofloat 33 glass (Figure 12). To estimate the thermal conductivity using the thermal model presented in Equation (6), the measurement results were normalized with respect to the measurement result obtained for a sensor on a thin-film membrane in ambient air.

In the case of the devices with AP 300/30 microstructures, the sensors were first measured under ambient conditions (AP 300/30) and then again on the custom-made vacuum chuck (AP 300/30 Vac). The thermal conductivities at both ambient and reduced pressure were then estimated using the previously derived thermal model (Equation (6)), and are presented in Table 2.

## 4. Discussion

The above results indicate that PowderMEMS microstructures are well suited to tailoring the heat propagation within miniaturized systems, for example, MEMS devices. For porous 3D-microstructures fabricated from silicon nitride and glassy carbon powder, thermal conductivity values such as those of Borofloat 33 substrates are achieved. The thermal conductivity measured for porous AP 300/30 microstructures is lower than any previously reported for inorganic materials that are compatible with MEMS processing. In comparison with glass and porous silicon, the two most widely used MEMS substrates for solid thermal isolation purposes, AP 300/30 microstructures provide a reduction in thermal conductivity by up to two orders of magnitude. By decreasing the gas pressure inside the PowderMEMS microstructure, thermal conductivities close to that of air ($\lambda_{Air}=0.026\;W/\mathrm{mK}$) can be achieved.

**Figure 13:**
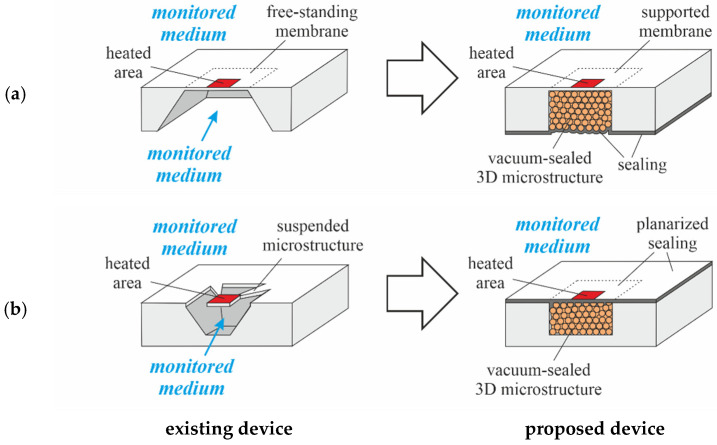
Modification of MEMS-based thermal sensors with a (**a**) free-standing membrane and (**b**) suspended microstructure using PowderMEMS technology. On the left side the conventional sensor design is shown (existing device), and on the right side a corresponding approach based on a vacuum-sealed 3D microstructure (proposed device) is presented.

PowderMEMS microstructures can be used in thermal MEMS such as IR detectors, gas sensors, or calorimetric flow sensors for the purpose of thermal isolation. Figure 13 illustrates the modification of two common types of MEMS-based calorimetric flow sensors with porous 3D microstructures. In both cases, the presence of a mechanically solid body would improve the resilience of the free-standing structures against overpressure events, in addition to suppressing vibrational eigenmodes that may result in aberrant sensor readings or outright mechanical failure. Additionally, in the case of membrane-based sensors (Figure 13a), parasitic effects within the back side cavity, caused by the convection of the monitored medium, would be suppressed. The proposed devices in Figure 13 represent two basic options to integrate PowderMEMS microstructures into MEMS. In Figure 13a, the porous 3D microstructure is manufactured at the very end of the MEMS process. To achieve a stable vacuum inside the porous microstructure, films having a thickness of several micrometers can be deposited by standard MEMS chemical and physical vapor deposition processes. It has already been shown that, for example, silicon oxide can be deposited pinhole-free by PECVD on top of a porous 3D microstructure [19,21]. However, the (long-term) hermeticity of such sealings remains to be investigated. A second approach is presented in Figure 13b. Here, the porous microstructure is first manufactured inside an etched microcavity and then sealed and planarized. First attempts at the planarization of such structures have been reported in [30].

**Figure 14:**
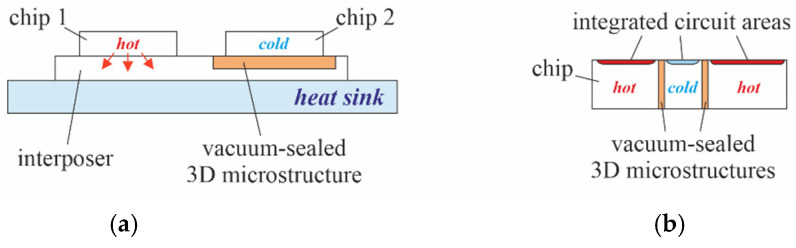
(**a**) Interposer substrate with integrated PowderMEMS structure on top of a heatsink for thermal isolation of chips, which are in close proximity. (**b**) Semiconductor substrate with PowderMEMS structures spanning its full height, creating thermally and electrically isolated islands within an integrated circuit.

Another field of application for PowderMEMS microstructures may be the creation of thermally isolated areas within a miniaturized system or an integrated circuit to separate “cold parts” and “hot parts”. The schematic cross-section in Figure 14a, for example, shows an interposer with chips at very different temperatures, assembled in close proximity.

The porous 3D microstructure beneath chip 2 protects it from the heat generated by chip 1. In Figure 14b, “through silicon” porous 3D-microstructures are used to create thermally isolated islands within a larger integrated circuit.

Additionally, it should be noted that, if non-conductive powders are chosen, PowderMEMS structures also provide galvanic isolation. To connect thermally separated parts of an integrated circuit as shown in Figure 14b electrically, thin-film metal traces can be routed across PowderMEMS structures [16].

## 5. Conclusions

This work describes a novel process for microscale thermal isolation by solid porous 3D microstructures. A process for the modification of existing thermal MEMS with thin-film membranes is presented. A model for the estimation of the resulting thermal conductivity of the porous microstructure was developed, and porous 3D microstructures made from three different powdered materials were created and investigated regarding their suitability for thermal isolation. Microstructures agglomerated from Aeroperl 300/30 were found to perform best. By lowering the residual gas pressure inside the structure, a further decrease in thermal conductivity was observed. The final thermal conductivities observed were close to 0.1 W/mK in ambient air and close to 0.04 W/mK for porous structures under vacuum.

## 6. Patents

Table 3 summarizes the patents related to this work.

## Figures and Tables

**Table 1:** Comparison of absolute values of $R_{Th}$ obtained by measurements (*n* = 5 for membrane and *n* = 4 for glass) and 3D FEM simulation.

Heater Substrate	$\mathrm{Measured}\;R_{Th}\;\left(K/W\right)$	$\mathrm{Simulated}\;R_{Th}\;\left(K/W\right)$	Error (%)
Unmodified membrane	7693.54±16.26	7716.66	0.3±0.21
Glass	1393.18±11.32	1292.32	7.2±0.75

**Table 2:** Estimation of $\lambda_{Por}$ for AP300/30 microstructures from normalized measurements (*n* = 5) of $R_{Th}$ using the model presented in Equation (6).

Gas Pressure	$\mathrm{Measured}\;R_{Th,norm}$	$\lambda_{Por}\;\left(W/mK\right)$
0.1 MPa (Ambient)	$0.675\pm 2.15\cdot 10^{-2}$	$0.098\pm 7\cdot 10^{-3}$
0.5 Pa (Vacuum chuck)	$0.916\pm 5.64\cdot 10^{-2}$	$0.039\pm 1\cdot 10^{-2}$

**Table 3:** Patents related to this work.

No.	Granted Patents	Short Description of the Patent Family
1	EP2670880B1 US9221217B2 JP6141197B2	Fabrication of porous 3D microstructures, basic method
2	EP3523637B1 US11137364B2	Thermal isolation based on porous 3D microstructures

## Data Availability

Not applicable.
